# Solid Immersion Facilitates Fluorescence Microscopy with Nanometer Resolution and Sub-Ångström Emitter Localization

**DOI:** 10.1002/adma.201203033

**Published:** 2012-09-12

**Authors:** Dominik Wildanger, Brian R Patton, Heiko Schill, Luca Marseglia, J P Hadden, Sebastian Knauer, Andreas Schönle, John G Rarity, Jeremy L O'Brien, Stefan W Hell, Jason M Smith

**Affiliations:** Department of NanoBiophotonics, Max Planck Institut for Biophysical ChemistryAm Fassberg 11, 37077 Göttingen, Germany; Centre for Quantum Photonics, Department of Electrical and Electronic Engineering & H. H. Wills Physics Laboratory, University of Bristol, Merchant Venturers BuildingWoodland Road, Bristol BS8 1UB, UK; Department of Materials, University of OxfordParks Road, Oxford OX1 3PH, UK

**Keywords:** nanoscopy, color centers, ODMR, solid immersion lens, STED

Standard far-field optical microscopy techniques provide non-invasive access to the interior of transparent samples, albeit with a resolution that is constrained to about half of the wavelength of light *λ*.^1^ By providing a resolution that is no longer limited by diffraction, emerging far-field optical nanoscopy or superresolution techniques are transforming the life sciences, but have also implications in the material and information sciences. Exemplifying the latter are concepts for quantum computation relying on point defects in a closely spaced crystal lattice forming coupled quantum systems. A candidate for such a system is the negatively charged nitrogen vacancy (NV) point defect in diamond, which consists of a lattice vacancy located next to a substitutional nitrogen. The NV center displays remarkable properties as a spin register, combining long coherence times at room temperature with convenient means for optical and microwave initialization followed by fluorescence-based read-out. Recent works have shown that the spin states of NV centers separated < 10 nm apart can communicate with each other on sub-microsecond time scales, which is sufficiently fast to envisage an array of entangled quantum systems as a crucial step towards a quantum processor.^2^, ^3^ Another application is the use of NV centers as optical sensors. Owing to their optically addressable spin state, these atom-like fluorescent defects can be used for magnetic and electric field metrology and bio-sensing.^4–7^ Clearly, their use will greatly benefit from, or even fully rely on, the possibility to record individual centers in densely packed clusters or arrays.

First approaches to achieve nanometer scale resolutions relied on AFM-like configurations featuring a NV-center at the scanning tip.^8^ But like all near-field scanning techniques these approaches are slow and limited to surfaces. Fortunately, NV centers in bulk diamond are photostable and thereby exceptionally well suited for far-field optical imaging with diffraction-unlimited resolution. It has been demonstrated that stimulated emission depletion (STED) microscopy, can image single NV-centers with a spatial resolution that is 10–20 times better than the diffraction limit.^9^, ^10^ While related techniques, such as ground state depletion microscopy have also approached this level, STED has maintained its pivotal role due to its ability to provide images as raw data, its outstanding signal-to-noise ratio, its recording speed, and its low demands on sample preparation.^11–13^

Here, we surpass previous limits for STED microscopy of NV centers in diamond, demonstrating that it provides a resolution down to 2.4 ± 0.3 nm in raw data images. This record far-field optical resolution is attained by focusing the STED beam through a solid immersion lens (SIL) fabricated into the diamond.^14–16^ Our data shows that the combination of STED and SIL should be highly effective to characterize arrays of coupled NV spins and to advance applications of NV centers in general, particularly their use as sensors of nanoscale magnetic fields.

STED microscopy separates features that are closer than the diffraction barrier by forcing them to fluoresce sequentially. Therefore, besides employing light for exciting emitters to their fluorescent state, this nanoscopy method uses a second beam, called the STED beam, that is so intense to keep emitters non-fluorescent by stimulated radiative de-excitation. At the same time, the STED beam features a zero so that features located at, or within a small distance *d/2* < *λ*/4 from the zero are still capable to fluoresce. Thereby the spatial distance within which markers can fluoresce simultaneously, is reduced to values *d* ≪ *λ*/2, a distance denoting the spatial resolution and scaling inversely with the square-root of the STED-beam's laser power *P_STED_*.^17^ For *P_STED_*→∞, *d* converges to zero, meaning that under perfect conditions *d* is just limited by the available *P_STED_* or by the extent of the defect itself (<0.5 nm). However, the strategy of maximizing the resolution by increasing *P_STED_* is challenged by aberrations or scattered light that prevent the formation of a true zero field of the STED beam. Correcting for these errors where possible, the smallest values of *d* that have been achieved beneath a planar diamond surface are 8 nm in two dimensions (2D) and 5.6 nm in a single dimension (1D) in the focal plane.^9^, ^10^ Clearly, any attempt to improve these values must address these limiting sources of aberration or scattering in the system.

One such aberration is due to the refractive index of diamond of *n* = 2.4 which poses multiple problems for imaging NV centers beneath a planar surface. Refraction and reflection caused by focusing with a high semiaperture angle (typically *α* = 68°) through the surface reduces the maximum intensity in the focal region and also the efficiency of fluorescence collection. Moreover, the spherical aberration imposed on the focusing when passing from the immersion medium of an immersion objective lens (typically oil with *n* = 1.5) into diamond causes lateral and substantial axial enlargements of the focal diffraction maximum in proportion to the penetration depth, reducing the intensity further. The reflections and aberrations are exacerbated when using air lenses.^18–20^

A technique that corrects both for losses and aberration is to use a solid hemispherical lens of diamond. All rays reach the diamond-air interface at normal incidence and so all aberration and refraction effects are eliminated with only a reflection of ≍17% remaining as a loss. Due to the emitter being embedded in a sample of *n* = 2.4, the SIL allows for an effective NA = *n* sin*α* that is greater than that of the dry or oil immersion lens being used. The NA asymptotically approaches *n* for increasing *α*, and with the *α* = 68° used here, we attain NA = 2.2. Additionally, the SIL acts to magnify the lateral image by a factor of *n_SIL_/n_Im_*, with *n_Im_* being the refractive index of the regular immersion medium of the lens. The resulting factor of 1.6 for an oil immersion lens is used to correct all the results in this paper.

An example of a confocal image of the diamond around the position of the SIL is shown in **Figure** **1**e. The single NV center situated underneath the SIL is clearly visible. As expected, this center is much brighter than the surrounding centers in the sample.

**Figure 1:**
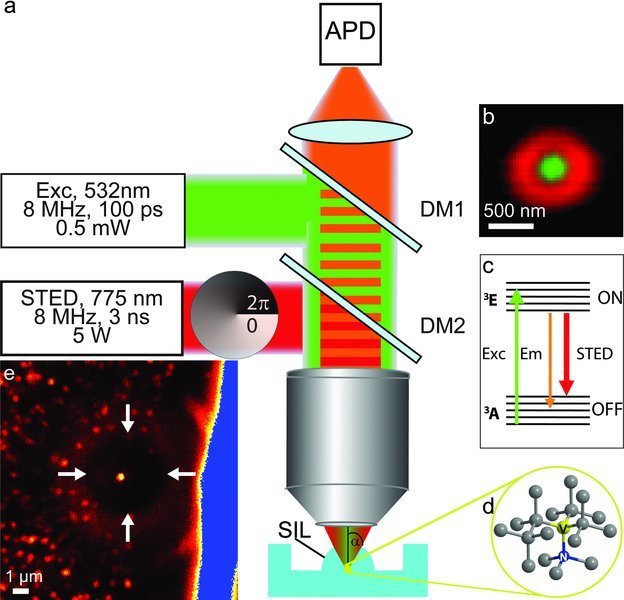
a) STED nanoscope for optical detection of magnetic resonances (ODMR), addressing individual diamond nitrogen vacancy (NV) centers inside a solid immersion lens (SIL) made of bulk diamond. STED nanoscopy is performed by combining a pulsed excitation (532 nm) and a pulsed STED (775 nm) beam with dichroic mirrors and focusing them into the sample using a high angle lens. The helical phase ramp imposed on the STED beam generates a doughnut-like intensity distribution at the focal plane. b) The measured excitation and STED beam focal light spots. c) Simplified excitation and emission energy scheme showing the basic transitions of the NV center d) Diamond lattice, showing the configuration of a single NV e) Confocal fluorescence image of the diamond sample without STED; the noticeable SIL periphery is indicated by arrows. The NV within the SIL is 5 times brighter than those beneath the surrounding planar surface. The scale bar applies to the fluorescence image as rendered in combination with the SIL.

Next, we imaged the NV center under simultaneous illumination with 532 nm and STED light. **Figure** **2** compares the images of this NV center in confocal (a,c) and STED (b,d) mode. At maximum STED laser intensity we reduced the range in the focal plane in which fluorescence was allowed to 4.2 nm and 6.8 nm, in the *x*- and the *y* direction, respectively. The values give the full-width-at-half-maximum (FWHM) of the function describing the probability of emission, which also represents the effective point-spread-function (E-PSF) of this far-field optical nanoscopy modality. The demonstrated resolving power amounts to an increase by ≍50-fold compared to standard (non SIL-STED) confocal microscopy of NV centers and to a 1.8-fold increase compared to previous STED realizations without a SIL.

**Figure 2:**
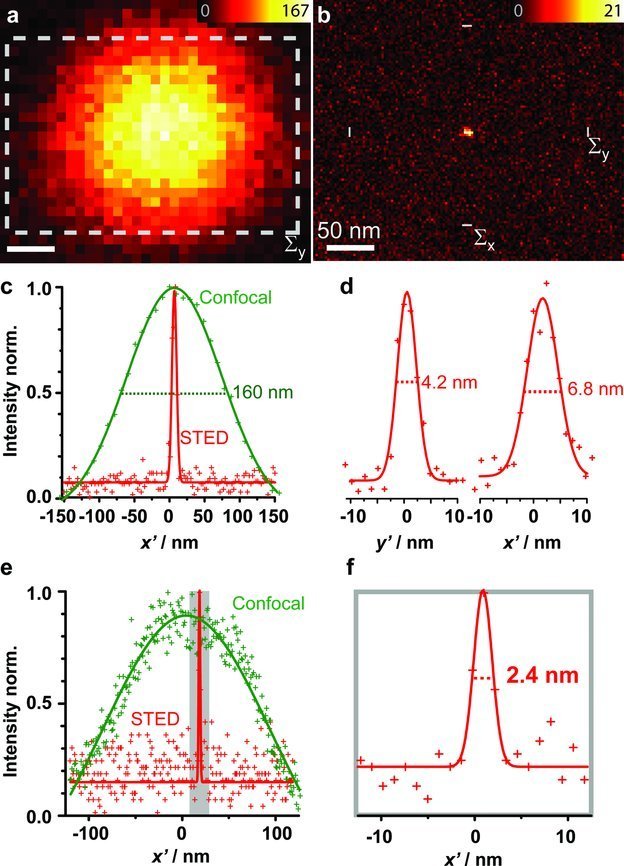
a) Confocal and b) STED image of a single NV located underneath a SIL yielding the effective point spread function (E-PSF) of the SIL-STED microscopy c) The SIL-enhanced confocal E-PSF has a FWHM of 160 nm. d) In the STED mode the FWHM of the E-PSF is reduced to 4.2 ± 0.1 nm (*y* direction) and 6.8 ± 0.2 nm (*x* direction). STED decreases the focal area in which the NV^−^ center is signaling by about 900-fold. The location of the imaged individual emitter can be given with even higher precision by calculating the centroid of its image; here it is established with 0.15 and 0.09 nm precision for *x* and *y*, respectively. e) and f) Intensity profiles showing the narrowing of the effective point-spread-functions and the concomitant improvement of resolving power with a line-shaped STED beam intensity zero oriented along the y-axis. e) The spatial extent (FWHM of the effective PSF) in which the NV is allowed to emit is reduced from 161 nm (confocal) to only 2.4 ± 0.3 nm (STED), as shown in the enlarged view of panel b. The data demonstrates a 67-fold resolution increase by STED, corresponding to 1/322 of the vacuum wavelength of the STED beam used. The position of the emitter is here localized with 0.33 nm.

Note that the location of the imaged individual emitter can be given with even higher precision by calculating the centroid of its image, a process known as localization. The localization precision scales inversely with the square root of the number of photons detected from the emitter. The precision with which we established the (central emission) coordinate of the NV center shown in Figure 2 was 0.15 nm and 0.09 nm for the *x* and the *y* direction, respectively.

The difference in FWHM between the *x*- and the *y*-direction can be explained by an azimuthally inhomogeneous intensity distribution around the central STED beam minimum. Additionally, the possibility of a small drift between successive x-oriented scan lines cannot be excluded, also contributing to the inequality in *x* and *y* FWHM measurements. The fluorescence still displayed a good signal to noise ratio at the highest STED beam intensity. Thus, one can still expect to achieve an even narrower E-PSF by using a phase filter that generates a line-shaped minimum in the focal STED beam, providing a steeper intensity gradient but a 1D resolution enhancement only. The results are shown in Figure 2e,f. With SIL-aided STED we achieved an E-PSF featuring a FWHM of 2.4 ± 0.3 nm, representing the highest resolving power demonstrated with far-field optical microscopy so far. Note that the 2.4 nm resolution value also exceeds that of near-field optical techniques.^21^ It is also worth noting that, due to the photostability of the NV centers and the possibility to locate the same NV repeatedly, it should also be possible to perform 1D measurements whilst rotating the line-shaped minimum around the optic axis after each scan, in order to record images with uniform 2.4 nm focal plane resolution.

Many applications of NV centers such as the optical detection of spin states and of magnetic fields have been limited by the spatial resolution and signal to noise ratios that require long acquisition times. Both challenges can be met through the use of SILs. In **Figure** **3**, we display typical optically-detected magnetic resonance (ODMR) measurements in a STED-SIL setting. In Figures 3a and b, an ESR spectrum is shown together with the applied pulse sequence: the sample is continuously illuminated with excitation light to simultaneously pump and read out the spin state of the NV center, whilst irradiating microwaves of varying frequency. A fixed magnetic field of a few mT is applied to provide a Zeeman splitting of the |*m_s_*| = 1 levels. When the microwave frequency is in resonance with a magnetic dipole transition between these levels and the *m_s_* = 0 level, the NV is driven between the bright *m_s_* = 0 state and one of the |*m_s_*| = 1 states which are ≍30% less bright. The net result is a fluorescence dip at the resonance frequency, indicating the strength of the local magnetic field.

**Figure 3:**
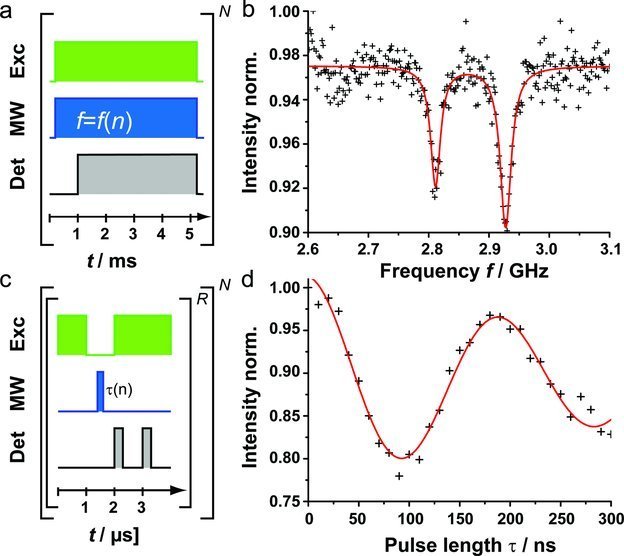
Optical detection of magnetic resonances of NV electron spins with SIL-STED. a) Sequence of optical excitation (Exc), microwave exposure (MW), and detection time window (Det) used for recording an b) electron spin resonance (ESR) spectrum. c) Corresponding sequence for inducing the Rabi oscillation shown in d).

Figure 3c shows a coherent manipulation of the spin of the NV by driving a Rabi oscillation. In this measurement, the NV electron spin is first pumped into the *m_s_* = 0 state by a pulsed excitation at 532 nm, followed by a microwave pulse of varying length and tuned to one of the resonance frequencies. Depending on the duration of the microwave pulse, the probability to transfer the NV into the state *m_s_* = 1 changes, which is probed afterwards by a read-out pulse of excitation light. The right part (Figure 3d) shows an average over many measurements. In all ODMR measurements, the increased collection efficiency due to the SIL shortens the acquisition times by 2.2 fold.

Yet, the combination of STED microscopy with a solid immersion lens reported herein has led to the sharpest E-PSF attained in a far-field optical system to date. The results also suggest that after further optimization and provided that the interaction of neighboring (excited) NV is weak enough, even single nanometer resolution is possible. Since the resolution still scales with *λ* of the STED beam (here 775 nm), resorting to shorter wavelengths will be another avenue. While STED on NV centers should be possible at least down to 680 nm, other quantum systems should be found as well, whose signaling capability can be modulated at shorter λ.

While our study was carried out with a SIL crafted into a bulk, we note that implementations using a free-standing SIL should also be possible. Such implementations will be suitable for NV-based sensing applications fields requiring the NV center to be brought close to the sample. In this case, the sample is placed right on top of the flat surface of the SIL, whereas the NV is located right beneath the surface and read out through the curved side. Our results also indicate that related techniques requiring lower intensities, such as RESOLFT microscopy has the potential to achieve similar resolution.^22^, ^23^ Hence, we expect our study to have a pivotal role in the quest for attaining single digit nanometer resolution in various settings. Thus far, the unique combination of high resolution and signal attained with SIL-STED opens the door to numerous spin-spin interaction studies. It is also particularly advantageous for experiments requiring cryogenic temperatures in which oil immersion lenses can not be used. Moreover, our SIL-STED study has now presented a route to addressing single bits in a future quantum computer based on NV centers. With a field of view of ≍10 μm^2^ and a resolution < 10 nm one can envisage addressing quasi two-dimensional arrays of about ≍10^5^ NV centers coupled by spin-spin interactions. Last but not least, since the 2.4 nm resolution demonstrated herein is not yet limited on conceptual grounds, future refinements of the diamond SIL should yield an even higher resolution. Altogether, these results underscore the arguably unexpected ability of far-field optics to image quantum systems with diffraction-unlimited resolution.

## Experimental Section

*STED-ODMR Setup*: The components of the setup utilized for our experiments (Figure 1) have been detailed elsewhere.^12^ The NV centers in the diamond are excited by a triggerable diode laser (Pico TA, Picoquant, Berlin, Germany) operating at 532 nm and providing pulses of ∼100 ps duration. Stimulated emission is induced by a frequency-doubled fiber laser (IPG Photonics, Oxford, USA) emitting at 775 nm at a repetition rate of ∼8 MHz and a pulse length of 3 ns. The STED beam passes through an optical glass plate (RPC Photonics, Rochester, NY, USA) imposing a 0–2π helical phase-ramp which generates the desired toroidally (doughnut) shaped intensity distribution in the focal region. The two beams are spatially superimposed by dichroic mirrors and focused by the objective lens (100x oil immersion, NA = 1.4, Leica, Wetzlar, Germany). Fluorescence emitted from the sample is collected by the same objective lens and filtered to block STED and excitation light. The remaining fluorescence is focused onto the core of a multimode fiber which acts as confocal pinhole, and is finally detected by an avalanche photodiode (Perkin & Elmer, Waltham, USA). Scanning the sample directly yields the (subdiffraction resolution) image as raw data.

*SIL Production*: The SILs were fabricated directly in the diamond by ablating the diamond with a focused beam of 30 keV gallium ions.^15^, ^16^ As the focal extent of the gallium beam is ≍10 nm in diameter, any residual surface roughness is much smaller than the wavelength range of the light involved (532–775 nm). We manufactured SILs ranging in size from 5–8 μm in diameter in two diamond samples. The first was a sample of high-purity polycrystalline diamond grown by chemical-vapor deposition. We directly milled several SILs of 5 μm diameter in this sample randomly placed on single crystallites. The density of single NVs in the sample is sufficiently high that a useful fraction of the SILs couple to individual or sparse ensembles of NV centers. One SIL in particular was found both to be coupled to a single defect center (as confirmed with single-photon antibunching measurements) and showed a 10-fold increase in detected fluorescence. The second sample was a high purity single crystal diamond in which regular arrays of NV centers had been generated at a depth of 4 μm by implantation of 6 MeV nitrogen ions followed by annealing at 800 °C. The 8 μm diameter SILs etched in this sample were registered to individual defects. Here, the best SIL showed an 8-fold increase in collected fluorescence. It is also worth noting that, while the SIL is small, the diamond-air interface for the SILs under investigation is 12–15*λ* from the emitter and so the solid immersion provided by the SIL maintains far-field optical conditions.
