# Supplementary material for: Solid Immersion Facilitates Fluorescence Microscopy with Nanometer Resolution and Sub-Ångström Emitter Localization
Source: Adv Mater. 2012 Sep 12;24(44):OP309–13. doi: 10.1002/adma.201203033 (PMC3546393; doi:10.1002/adma.201203033)
Supplement: Supplementary file 1 [file adma0024-0000-SD1.pdf]

Copyright WILEY-VCH Verlag GmbH & Co. KGaA, 69469 Weinheim, Germany, 2012.

ADVANCED MATERIALS

Supporting Information

for *Adv. Mater.*, DOI: 10.1002/adma.201203033

Solid Immersion Facilitates Fluorescence Microscopy with Nanometer Resolution and Sub-Ångström Emitter Localization

Dominik Wildanger, Brian R. Patton, Heiko Schill, Luca Marseglia, J. P. Hadden, Sebastian Knauer, Andreas Schönle, John G. Rarity, Jeremy L. O'Brien, Stefan W. Hell,* and Jason M. Smith*

Supporting Information:

Solid immersion facilitates fluorescence microscopy with nanometer resolution and sub-Ångström emitter localization

By *Dominik Wildanger**, *Brian R. Patton*, *Heiko Schill*, *Luca Marseglia*, *J.P. Hadden*, *Sebastian Knauer*, *Andreas Schönle*, *John G. Rarity*, *Jeremy L. O'Brien*, *Stefan W. Hell** and *Jason M. Smith*

Dr. Dominik Wildanger, Dr. Heiko Schill, Dr. Andreas Schönle, Prof. Stefan W. Hell
Max-Planck-Institut for Biophysical Chemistry
Am Fassberg 11
37077 Göttingen, Germany

Dr. Brian R. Patton, Dr. Luca Marseglia,. J.P. Hadden, Sebastian Knauer, Prof. John G. Rarity, Prof. Jeremy L. O'Brien
Centre for Quantum Photonics, Department of Electrical and Electronic Engineering & H. H. Wills Physics Laboratory,
University of Bristol,
Merchant Venturers Building, Woodland Road
Bristol BS8 1UB, UK

Dr. Jason M. Smith
Department of Materials, University of Oxford,
Parks Road,
Oxford OX1 3PH, UK

*E-mail: dominik@wildanger.net,
*E-mail: hell@nanoscopy.de

Fluorescence off-switching

To gain a better quantitative understanding of the effect of the SIL on the imaging, first we assessed how well the SIL enhanced the fluorescence inhibition, i.e. we recorded a depletion curve quantifying the depletion of the fluorescent state as a function of the STED beam power. To this end, we removed the helical phase ramp from the STED beam path, leaving a nearly Gaussian intensity distribution in the focal plane. The fluorescence from the sample is then measured as a function of the applied STED beam

power while keeping the power of the 532 nm excitation beam constant. Since the threshold (saturation) intensity I_S is an intrinsic property of the NV center, a steeper depletion curve within the SIL can only be explained by a local intensity increase through better focusing, i.e. a smaller focal area at the defect. The results shown in figure S1 confirm the SIL to be performing as desired.

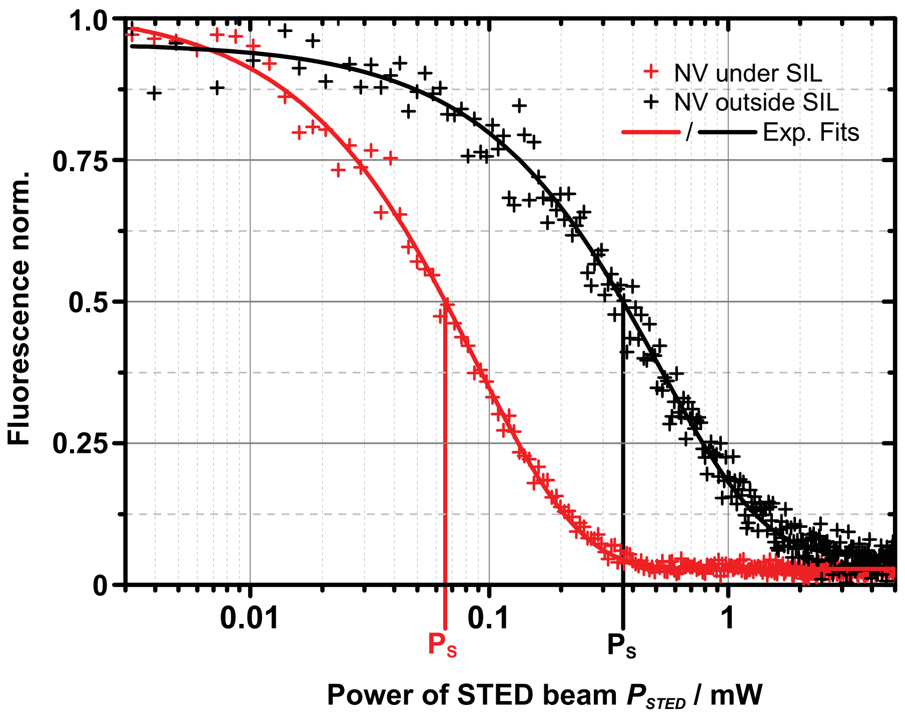

Figure S1 Inhibition of the NV fluorescence signal by a Gaussian STED beam (i.e. depletion curve) for equivalent NV centers under a SIL (red) and under a planar surface (black). Pulsed excitation light focused by a 1.4 numerical aperture lens was fixed in power and superimposed with a regularly focused STED beam. The fluorescence was recorded as a function of the time-averaged power of the pulsed STED beam measured in the back aperture of the lens. The power value P_S corresponding to the 50 % threshold/saturation intensity I_S is shown to be 61 µW with SIL and 359 µW without.

Multiple NV

So far all experiments reported were carried out on a single NV center situated almost at the center of a SIL. To prove the suitability of SIL-STED for the investigation of spin-

spin-interactions and to explore to which extent aberrations at the boundaries of the SIL limit the imaging quality, we imaged clusters of NV centers which were situated 1.1 µm away from the center of one SIL (figure S2b). We found that aberrations due to SIL imperfections for NVs observed close to the edge of the SIL field of view did not appear to affect the imaging substantially. Two neighboring NVs which could not be resolved in confocal mode are separated in the STED image. As it is known that the spin-lifetime is unaffected by the fabrication processes involved in SIL manufacture, the spins should be individually addressable in optical recordings with subdiffraction resolution.

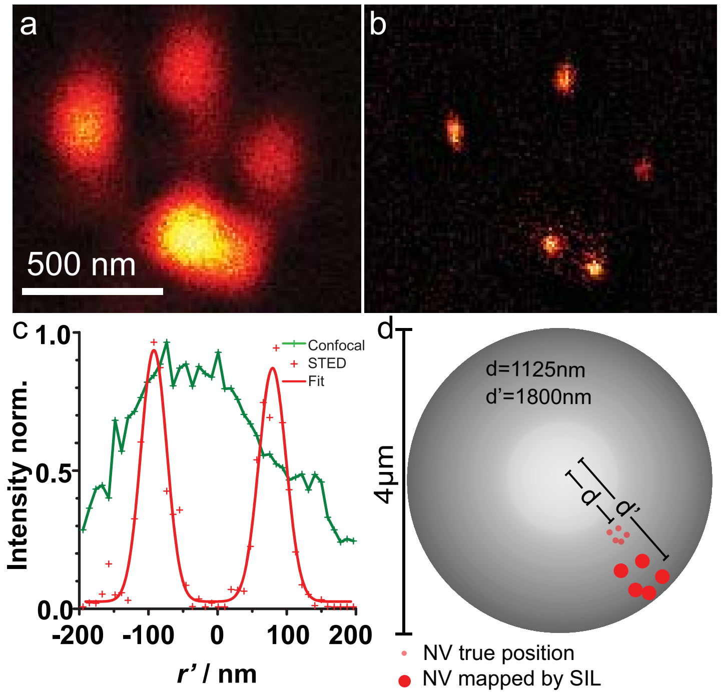

Figure S2: Imaging of NV groups with subdiffraction spacing. (a) Confocal image of multiple NV centers within a SIL; (b) STED image of the same NV group (background subtracted); (c) Line intensity profile of the lower pair of NV centers of panels a and b quantifying the resolution in the corresponding confocal and STED data. d) Comparison of the actual position of the group with its apparent position due to the magnification caused by the SIL. Although the imaging is ≈1.1 µm away from the SIL's center, the STED image remains clear, exhibiting little aberration.

SIL-STED with air objective lens

In almost all STED microscopes, high NA oil- or water-immersion objective lenses have been used which cannot operate under vacuum or cryogenic conditions. However, many quantum experiments carried out with the NV spin system require cryogenic conditions. Therefore, we investigated the efficacy of high angle air objective lenses for addressing NV centers with SIL-STED. As shown in figure S3, using a dry lens of α=65° , we achieved d=7.6 nm, i.e. a demonstrated potential to resolve well beyond the 10 nm (corrected by the magnification factor of 2.4) benchmark required for investigating spin-spin interactions. Hence, our approach of combining SIL with STED should facilitate low temperature studies of spin-spin interactions.

There are several reasons why the resolution attained with air lenses was slightly lower than with oil immersion counterparts, with one being the central minimum of the doughnut. Usually the doughnut shape of the STED beam is prepared by scanning a gold bead across the focus and optimizing for the least amount of scattered light stemming from the doughnut minimum. In the case of an immersion system, such optimization is facilitated by the fact that the gold bead is immersed in a liquid whose refractive index (~1.5) matches that of the glass plate onto which the bead is mounted. In the case of a dry lens, such matching is not possible, meaning that the bead signal is overlapped by light scattered from the mounting plate. Additionally, the SILs under investigation are known not to be perfectly hemispherical, which also implies aberrations filling up the central zero.

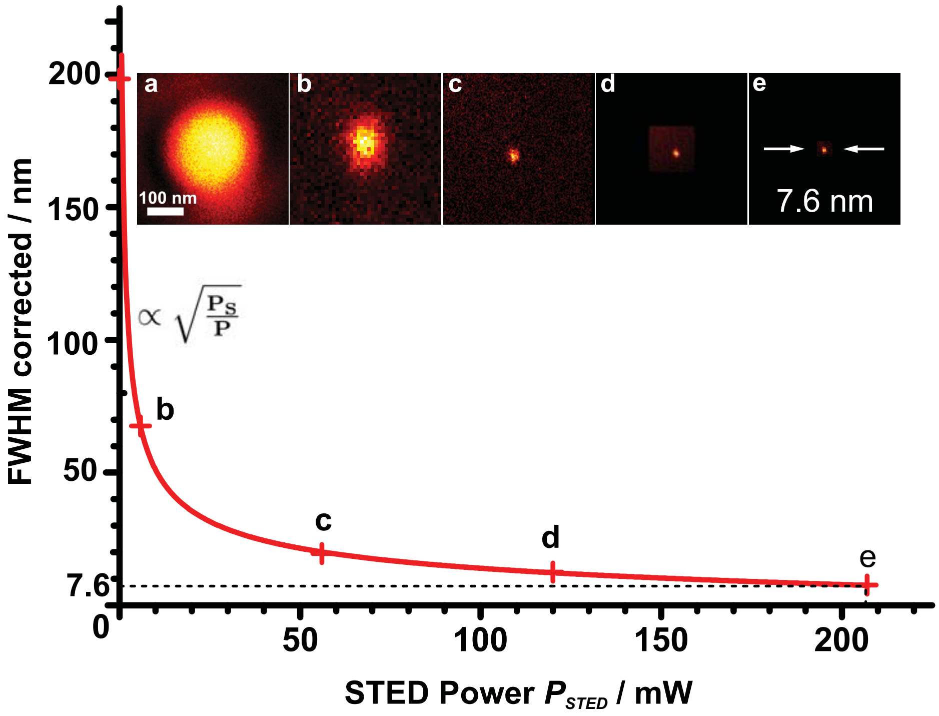

Figure S3: SIL-STED imaging of NV centers with an air objective lens. The images of these point defects show how the resolution (FWHM of the effective PSF), increases with the STED intensity down the single nanometer range, following an inverse square-root law.
